# 

**Published:** 1887-06

**Authors:** 


### THE AMERICAN DENTAL ASSOCIATION.

*Chicago, June 25th, 1887.*

·All railroads, west of Chicago, and from Chicago leading to Niagara Falls, will carry members and delegates of the American Dental Association at one and one-third fares.

READ THE RULES CAREFULLY.

Each delegate must purchase a first class ticket to the place of meeting, for which he will pay the regular fare and upon request ticket agent will issue to him a certificate of such purchase.

If through tickets cannot be procured at the starting point, delegates will purchase to the most convenient point where such through tickets can be obtained and re-purchased through to place of meeting, requesting a certificate from the ticket agent at the point where the re-purchase is made.

Tickets for the return journey will be sold by the ticket agents at the plaee of meeting at one third the highest limited fare, only to those holding certificates signed by the ticket agent at point where through ticket to place of meeting was purchased, and countersigned by the Secretary or clerk of the convention, certifying that the holder has been in attendance upon the convention. Tickets are good, going three days before the meeting and returning, three days after its termination.

A. W. HARLAN,
*Chairman Executive Committee.*

P. S.—Equally favorable rates are expected from the South and East.

---

### VIRGINIA STATE DENTAL ASSOCIATION.

### PRESIDENT'S OFFICE.

*Fredericksburg, Va., April 28, 1887.*

The Eighteenth Annual Session of our State Association will be opened at the Hygeia Hotel, Old Point Comfort, Tuesday, August 29, 1887, at 10 o'clock A. M.

You are cordially invited and urged to be present at what we expect to be the largest and most enthusiastic meeting of our profession ever held in the South.

The Southern Dental Association will meet at the same place, on the 29th, by our invitation, and we desire that every dentist in the State should be present to extend to our guests a genuine Virginia welcome.

The attention of the members of committees is particularly called to Article VI., Sections 2, 3 and 4, of the Constitution, a copy of which will be found herewith. Arrangements will be made for publishing the papers in the *Southern Dental Journal.*

Full particulars and details will be given in future circulars. In the meantime make all your arrangements with a view to being present.

GEO. H. CHEWNING, *President.*

### SOUTHERN DENTAL ASSOCIATION.

The annual session will be held at Old Point Comfort, (Fortress Monroe) Virginia, in the Hygeia Hotel, beginning August 30th.

W. W. H. THACKSTON, M. D., D. D. S., PREST.

FARMVILE, VA.

EXECUTIVE COMMITTEE.

DR. J. HALL MOORE, Richmond, Va., Chairman.
DR. J. R. WOODLEY, Norfolk, Va.
DR. E. S. CHISHOLM, Tuscaloosa, Ala.

COMMITTEE OF ARRANGEMENTS.

DR. J. R. WOODLEY, Norfolk, Va., Chairman.
DR. T. S. PARRAMOR, Hampton, Va.
DR. G. S. F. WRIGHT, Columbia, S. C.

### AMERICAN DENTAL ASSOCIATION.

CHANGE OF PLACE OF MEETING.

The twenty-seventh annual meeting of the American Dental Association will be held at Niagara Falls, commencing Tuesday, August 2d, 1887.

GEO. H. CUSHING, *Rec. Secretary.*

### THE NATIONAL ASSOCIATION OF DENTAL EXAMINERS.

*Orange, N. J., June 21st., 1887,*

The annual meeting of the National Association of Dental Examiners will be held at Niagara Falls on Monday, August 1st, 1887, at 3 P. M.

FRED. A. LEVY, *Secretary.*

### *To Readers and Correspondents!*

Communications intended for publication, and exchange journals and papers should be addressed to the EDITOR OF THE AMERICAN JOURNAL OF DENTAL SCIENCE, 845 N. Eutaw St., Baltimore, Md. All letters relating to the business of the JOURNAL, or containing cash remittances, to be directed to Messrs. SNOWDEN & COWMAN. Articles for publication should be received by the editor at least three weeks previous to the first month on which the number in which it is designed for them to appear, is issued.

☞ The AMERICAN JOURNAL OF DENTAL SCIENCE will contain fifty pages of reading matter in each number, making a volume of about *Six Hundred* pages,

EXCLUSIVE OF ADVERTISEMENTS.

